# Prevention of Hepatorenal Insufficiency Associated with Lead Exposure by *Hibiscus sabdariffa* L. Beverages Using In Vivo Assay

**DOI:** 10.1155/2022/7990129

**Published:** 2022-02-10

**Authors:** Samah A. El-Hashash, Mohamed A. El-Sakhawy, Eman E. El-Nahass, Mohamed A. Abdelaziz, Walid Kamal Abdelbasset, Mona M. Elwan

**Affiliations:** ^1^Department of Nutrition and Food Science, Faculty of Home Economics, Al-Azhar University, Nawag, Tanta City, P.O. Box 31732, Egypt; ^2^Department of Medical Laboratory Sciences, College of Applied Medical Sciences, Prince Sattam Bin Abdulaziz University, Al-Kharj 11942, Saudi Arabia; ^3^Department of Medicinal and Aromatic Plants, Desert Research Center, Cairo, Egypt; ^4^Department of Zoology, Faculty of Science, Tanta University, Tanta City, Egypt; ^5^Department of Basic Medical Sciences, College of Medicine, Prince Sattam Bin Abdulaziz University, Al-Kharj 11942, Saudi Arabia; ^6^Department of Medical Physiology, College of Medicine, Al-Azhar University, Cairo, Egypt; ^7^Department of Health and Rehabilitation Sciences, College of Applied Medical Sciences, Prince Sattam Bin Abdulaziz University, Al-Kharj 11942, Saudi Arabia; ^8^Department of Physical Therapy, Kasr Al-Aini Hospital, Cairo University, Giza, Egypt

## Abstract

Lead pollution is a major environmental challenge worldwide. Therefore, dietary interventions that are aimed at preventing lead's deleterious effects on body organs are needed. The study's goal was to study and compare the protective effect of cold and hot beverages of Roselle (*Hibiscus sabdariffa* L.) red calyces (CRB and HRB, respectively) on liver and kidney insufficiency associated with lead exposure in male rats. Adult albino rats (32 males) were divided into four groups of equal number, including a normal control (group 1), while groups from 2 to 4 received lead acetate (20 mg/kg body weight/day) and were kept untreated (group 2). The 3rd and the 4th groups received CRB and HRB (0.5 ml/100 g body weight/day), respectively, for 6 weeks. The gain in the body and relative weights of the liver and kidneys were calculated. Liver and kidney functions were determined in serum, while lead, delta-aminolevulinic acid dehydratase, and oxidative stress markers were established in tissues. Specimens from the liver and kidney of sacrificed rats were histopathologically examined. The total activity of antioxidants and total content of anthocyanin of both beverages were determined. Lead exposure resulted in its accumulation in tissues, leading to overweight and liver and kidney insufficiency along with oxidative stress, which was further confirmed by histological staining. CRB was more efficient than HRB in preventing the deleterious effects of lead intoxication. Due to their antioxidant properties, the present study proved that Roselle red calyx beverages, particularly the cold ones, are protective agents against lead-associated disorders in a rat model.

## 1. Introduction

Lead (symbol Pb) is a high toxic heavy metal that is not essential in living organisms. It is a widespread environmental pollutant used in acid battery and gasoline industries [1]. Lead is thought to be a multitarget toxicant, with oxidative stress being one of the most likely mechanisms of lead toxicity [2]. Pb exposure is associated with multiple ailments including brain and neurology defects, decreased attention and autism in children, birth defects, hypertension, high bone turnover, and hepatic and renal damages [3].

*Hibiscus sabdariffa* L. (*Hs*), also identified as Roselle, is one of the aromatic and medicinal plants belonging to the Malvaceae family; calyces vary in color from white-yellow to dark red, and that is attributed to the anthocyanin content. Worldwide, red variations control the Roselle markets, while white and light-red variations are mostly found locally. In Egypt, the red dehydrated calyces of *Hs* are utilized to prepare cold beverages and infusions, widely known as Karkadeh. In general, *Hs* is evaluated as a source of a large number of bioactive compounds including phenolic compounds, for instance, anthocyanins (e.g., delphinidin-3-O-sambubioside), phenolic acids (chlorogenic acid), and flavonols (quercetin and kaempferol derivatives). It is also rich in *α*-tocopherol and organic acids (the quinic acid as the primary). These composites are responsible for the various health-promoting properties of *Hs* which include antiobesity, hypolipidemic, anticarcinogenic, hypotensive, diuretic, antioxidant, and antimicrobial effects [4].

Since lead pollution is considered a major environmental health problem in Egypt and studies indicated that high concentrations of Pb were found in different types of Egyptian foods, either of the plant (14.4 *μ*g/g of spices and medicinal plants, 0.013–0.281 mg/kg in legumes, and 0.116–0.398 mg/kg in cereals) or animal (0.330 and 0.334 mg/kg in preserved meat of chicken luncheon and corned beef, respectively) origin [5–8], besides its presence in air and water, dietary interventions that are aimed at alleviating the harmful effects of lead on body organs are needed. The study's objectives were to investigate and compare protective effects of both cold and hot beverages of Roselle red calyces on liver and kidney injuries in lead-intoxicated experimental animals (rats).

## 2. Material and Methods

### 2.1. Plant Materials and Preparations

#### 2.1.1. Plant Materials

Roselle (*Hibiscus sabdariffa* L.) as dried red calyx was obtained locally from Tanta City's markets for medical herbs and plants, Al-Gharbiyah, Egypt. The herb was identified by Cairo University's Department of Botany Herbarium, Faculty of Science.

#### 2.1.2. Plant Preparations

Dry red calyces were finely ground using a hammer mill. After that, they were sieved by a two-millimeter pore size screen, stored in dry closed glass jars, and kept in the dark (at room temperature) until used. The traditional methods were followed in preparing both cold and hot Roselle beverages. In detail, cold Roselle beverage (CRB) was prepared by putting the Roselle powder in a suitable jar, followed by adding distilled water (25°C) to it; then, the jar was covered and refrigerated overnight (12 hr). After that, the obtained beverage was filtered. On the other hand, hot Roselle beverage (HRB) was prepared by putting the Roselle powder in a suitable jar, followed by pouring hot distilled water (95°C) over it; then, the lid was put on loosely to infuse them in the water solution for 15 min, and the obtained liquid was filtered. Both beverages were prepared daily without sugar or other sweeteners. The used dried calyx of Roselle to water ratio (1 : 40 *w*/*v*) was found to be appropriate for a drink with comparable intensity color to marketable produces [9].

### 2.2. Chemicals and Reagents

Casein (>80% protein), cellulose, choline chloride, DL-methionine, lead acetate minerals, sucrose, vitamins, and other scientific chemicals were acquired from El-Gomhouria Co., Cairo, Egypt. Biochemical kits were obtained from Biodiagnostics and Gamma Trade Companies for chemicals, Cairo, Egypt. Soybean oil and cornstarch were acquired commercially from Al-Gharbiyah Governorate, Egypt.

### 2.3. Determination of Biochemical Content

#### 2.3.1. Total Anthocyanins

Total anthocyanin determination was defined by the pH-differential technique [10]. These assay bases are depending on shifts towards low pH values by the chemical balance between flavylium cation (red color) and hydrated hemiketal (colorless) forms of the various anthocyanins.

Each sample was split into two aliquots, one with 400 mM NaCH_3_COO buffer (pH 4.5) and the other with 250 mM of KCl solution (pH 1.0). They were incubated at room temperature for 15 minutes after being mixed with each buffer to allow the reaction to reach equilibrium. Using a Varian Cary 50 Bio UV-Vis spectrophotometer (Italy), absorbance (Abs) was then measured in disposable cells (path length, one centimeter) at 510 and 700 nanometers. The instrument was set to zero using distilled water. To calculate total anthocyanin content, the following equation was used:

(1) $$\begin{array}{c} \text{TAC}=\frac{\text{Abs}\times \text{MW}\times \text{DF}\times 1000}{\varepsilon \times \text{l}}. \end{array}$$

**Figure 1:**
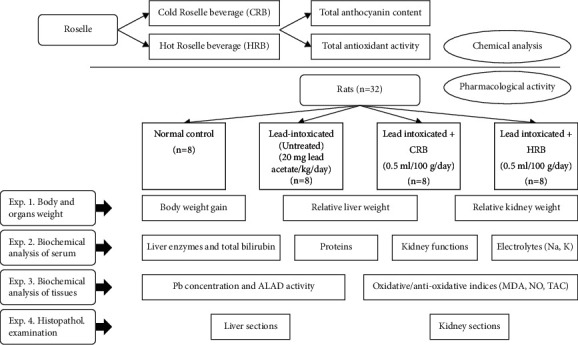
Graphical flow chart showing the experimental design of chemical analysis and pharmacological activity.

Abs = (Abs510 − Abs700) pH 1.0 − (Abs510 − Abs700) pH 4.5, MW means the molecular weight (449.2 g/mol) of cyanidin-3-glucoside, and *ε* means the molar absorptivity (26900 M^−1^ cm^−1^) of cyanidin-3-glucoside, too. DF is a dilution factor and a number of 1000 is an element for converting grams to milligrams, and 1 is the cell path's length (one centimeter). Results were presented as milligrams of cyanidin-3-glucoside equivalent/100 ml (mg C3G/100 ml) (Figure 1).

#### 2.3.2. Antioxidant Activity

To quantify antioxidant activity of Roselle beverages, the 2,2-diphenyl-1-picrylhydrazyl (DPPH) radical assay was used to investigate the scavenging activity measured by Villa-Rodríguez et al. [11]. By preparing a stock reagent solution; dissolving DPPH radical (2.5 mg) is dissolved in 100 ml of 100% methanol to make a stock reagent solution. Using a UV–Vis spectrophotometer, at 515 nm, the absorbance range was modified to 0.70 ± 0.02 (time 0). At 515 nm, the sample (10 *μl*) was mixed with DPPH (140 *μl*) solution and incubated in the dark for thirty minutes (*T* = 30); and the absorbance was then measured. DPPH inhibition calculation in percentages was performed with the following equation:

(2) $$\begin{array}{c} \%\text{of DPPH inhibition}=\left[\frac{{\text{Abs}}_{\text{time}0}-{\text{Abs}}_{\text{time}30}}{{\text{Abs}}_{\text{time}0}}\right]\times 100. \end{array}$$

Water-soluble analog of vitamin E (Trolox) synonymous to 6-hydroxy-2,5,7,8-tetramethylchroman-2-carboxylic acid has been utilized as the assay's average, and the result was presented as *μ*mol (Trolox/100 mil).

### 2.4. Pharmacological Activity

#### 2.4.1. Experimental Animals

Thirty-two healthy Albino (150 ± 5 g male) Sprague Dawley rats were sourced from an animal colony, Helwan Farm, VI Org., Cairo, Egypt.

#### 2.4.2. Preparation of Experimental Diet

Basal diet was formulated using fine ingredients. Each 100 g of the formulated basal diet consisted of 14, 4, 5, 3.5, 1, 0.25, 0.3, and 10 g of casein, soybean oil, cellulose, mineral mixture, vitamin mixture, choline chloride, DL-methionine, and sucrose, respectively, while corn starch was added up to 100 g [12]. These constituents were mixed well and sieved with a 2 mm (pore size) screen and stored in the dark until used at room temperature.

#### 2.4.3. Animal and Study Designs

Animals were kept in well-aerated cages under aseptic conditions in a room maintained at suitable humidity (23 ± 2°C) and every twelve-hour shift light : dark (LD) cycle and fed one week on basal feed for modification and adaptation. Later, experimental animals were weighed and separated into four groups (8 rats of each group). The first group was kept as a normal control group and fed on basal diet only, while groups from 2 to 4 were administered lead acetate (20 mg/kg body weight/day) [13]. At the same time, group 2 was fed on basal diet only, while the 3^rd^ and the 4^th^ groups were fed on basal diet and received CRB and HRB, respectively (0.5 ml/100 g body weight/day). Male rats received lead acetate, CRB, and HRB orally by a stomach tube all over the experimental period (protective study). No signs of scattering such as vomiting were noticed; i.e., the used dosage was suitable for each rat. Meanwhile, the experiment lasted for 6 weeks, ad libitum feeding (diet and water) was applied, and body weights were documented one time/week. By change of the body weight of each rat, its beverage volume was also changed (Figure 1).

#### 2.4.4. Tissue and Blood Samples

The blood samples were placed in dry and clean tubes after being collected from each rat's aorta. At room temperature, the serum of the blood was separated carefully by centrifugation (3000 RPM) for 10 minutes; after that, it was transferred into Eppendorf tubes and kept frozen at −20°C until laboratory testing. Before finishing the experiment, rats were fasted overnight and weighed before being anesthetized using Anahal and sacrificed. Moreover, livers and kidneys were removed by careful dissection, washed by frosted (1–4°C) saline (NaCl 0.9 g/100 ml), dried using filter paper, and weighed. After that, the specimens from each liver as well as the right kidney were stored at −20°C and −80°C for Pb determination and homogenate preparation, respectively, while a specimen from each liver as well as the left kidney was immersed in a buffered neutral formalin solution (10%) for latter histopathological examination. In general, all animal procedures were reviewed and approved by the Faculty of Science, Tanta University's Animal Ethics Committee (IACUC-SCI-TU-0234), based on the National Committee for Research's code of practice for the care and use of animals for scientific purposes.

#### 2.4.5. Total Body and Relative Organ Weights

An increase in body weight or gaining body weight expressed as BWG was measured by each rat's initial beginning from its finishing (final) weight. On the other hand, the relative weights of the liver and kidney (RLW and RKW) were calculated according to the following equation [14]:

(3) $$\begin{array}{c} \text{Relative organ weight}\left(\text{g}/100\text{ g}\right)=\left[\frac{\text{Organ weight}\left(\text{g}\right)}{\text{Final body weight}\left(\text{g}\right)}\right]\times 100. \end{array}$$

#### 2.4.6. Homogenization of Liver and Kidney Tissues

In order to prepare the liver tissue homogenate, tissues of the liver (1 g) were homogenized in ice-cold solution of KCl (1.15 g/100 ml) in 50 mmol/l buffer potassium phosphate solution (pH 7.4). For the preparation of kidney tissue homogenization, 500 mg of each kidney tissue was homogenized in five milliliters of phosphate buffer (0.1 M, pH 7.4). Ultrasonic Sonicator 4710 (the Cole-Parmer Inst. Company, USA) was used for homogenization. The homogenates were cooled down to (4°C) and centrifuged for 5 min at 4000 RPM. The supernatant was collected and preserved at −80°C for latter biochemical analysis.

### 2.5. Measurement of Biochemical Parameters in Liver and Kidney Tissues

#### 2.5.1. Lead

Tissue samples (about 500 mg), previously stored at −20°C, were put in a container (made of Teflon) with CHNO_3_ (seven milliliters) and hydrogen peroxide (one milliliter) and mineralized (Milestone STARTD, SK-10T, Sorisole, Italy, Milestone Srl). Milestone's recommendations were followed for the digestion process. Lead concentrations in tissues were determined using atomic absorption spectrophotometry (Agilent Technologies, Santa Clara, California, USA, AAS GTA 120 Graphite Tube Atomizer, 200 series AA). Meanwhile, the accuracy was validated with the referenced material 1577c-Bovine liver (LGS Standard, UK).

#### 2.5.2. Delta-Aminolevulinic Acid Dehydratase

The protein content of kidney and liver tissue homogenates was measured spectrophotometrically following Lowry et al.'s method [15] and using the albumin standard from bovine serum. Delta-aminolevulinic acid dehydratase (ALAD) activities in kidney and liver tissue homogenates were then determined following the method described by Fujita [16].

#### 2.5.3. Oxidant/Antioxidant Biomarkers

In liver and kidney tissue homogenates, nitric oxide, total antioxidant capacities (TAC), and peroxidation markers of the lipids (malondialdehyde, MDA) were identified following referenced methods [17–19].

#### 2.5.4. Liver Function Biomarkers

In serum, the level of total bilirubin was measured based on Schmidt and Eisenburg [20]. In addition, the enzyme activities in the liver, such as alkaline phosphatase (ALP), aminotransferases (ALT and AST), and gamma-glutamyl transferase (GGT), were determined [21–23].

#### 2.5.5. Kidney Function Biomarkers and Serum Electrolytes

Urea and creatinine have been determined in serum [24, 25]. For determination of serum sodium and potassium, the method described by Külpmann was followed [26].

#### 2.5.6. Serum Proteins

Total protein (TP) and albumin were determined following referenced methods [27, 28]. In addition, the distinction between serum total protein and albumin contents was calculated as serum globulin content.

### 2.6. Histopathological Examination

Tissue sections of the liver and kidney were immediately collected after sacrificing animals under appropriate anesthesia with Anahal and then were sliced into small pieces and fixed in 10% formalin for 24 hours. After washing, to remove the excess of fixative, the tissue samples were dehydrated in ascending serial ethanol and cleared by using xylene. Tissue slices were embedded in paraffin wax. Sections of 5 *μ*m thickness were mounted and stained with haematoxylin and eosin (H&E) for histological examination [29]. Slides were prepared for each organ. All microscopically examined slides were examined for histopathological changes.

## 3. Statistical Analysis

All collected data have been analyzed by using statistical software for windows (SPSS v. 20, Armonk, NY, USA). All measures have been presented as mean ± standard deviation. The data analysis has been performed utilizing the one-way ANOVA test. The significance between mean differences has been examined utilizing Duncan's test. The significant level has been set at *P* < 0.05.

## 4. Results

### 4.1. Total Anthocyanin Content and Total Antioxidant Activities of Roselle Beverages

The findings are illustrated in Table 1, indicating that cold Roselle beverage was higher in both anthocyanin content and antioxidant activity than the hot beverage.

### 4.2. Effect of Lead Exposure versus Roselle Beverages

#### 4.2.1. Effect on Body Weight Gain and Relative Liver and Kidney Weights

The effects of Roselle calyx beverages on body weight gain as well as absolute and relative weights of the liver and kidney in lead-intoxicated versus normal rats are illustrated in Table 2. At the beginning of the experiment, nonsignificant changes were observed in the body weight of groups experimented, while at the end, body weight gain of the untreated lead-exposed group was found to be significantly (*p* < 0.05) higher than that of normal values recorded by the control group. Both cold and hot beverages of Roselle red calyces (CRB and HRB, respectively) induced significant decreases compared with the untreated lead-exposed group; however, CRB was so effective that it was able to restore BWG to its normal value. At the same time, absolute weights of the liver and kidneys were not affected significantly by lead exposure or Roselle beverage administration. In contrast, relative weight of the liver was decreased significantly upon exposure to lead acetate and Roselle beverages could not induce significant changes despite the noticed elevation. As for relative kidney weight, it was not affected significantly as a result of lead exposure; however, the CRB-administered group exhibited significant increase compared with the HRB-administered one.

#### 4.2.2. Effect on Lead, ALAD, and Oxidative Stress Markers in Liver and Kidney Tissues

It could be noticed that regular exposure to lead acetate resulted in a significant (*p* < 0.05) rise in tissue Pb. Both CRB and HRB lowered Pb concentrations significantly in the studied tissues, with no significant differences regarding Pb concentrations in the kidneys. In contrast, liver Pb concentration in the CRB-administered group was the nearest to that of the normal control group. In contrary to Pb concentration, ALAD activity recorded a significant decrease in liver and kidney tissue homogenates of the untreated Pb-intoxicated group compared to the normal control group. Both CRB- and HRB-administered groups recorded significant elevation in ALAD activity in the studied tissues, but the enzyme activity levels of the CRB-administered group were the nearest from those of the normal control group. Moreover, while the untreated lead-intoxicated group recorded a significant increase in MDA concentrations, it recorded significant reductions in both nitric oxide and total antioxidant capacity in kidney and liver tissue homogenates as compared to the normal control group. Significantly, MDA, NO, and TAC in liver tissue homogenate (L. MDA, L. NO, and L. TAC) of Roselle calyx hot tea-administered group were the same as those of the untreated lead-intoxicated group, while the cold tea-administered group showed no significant differences in comparison to the normal control group. In contrast, both beverages improved the antioxidant defense system in the kidney tissue homogenate, as they increased TAC significantly not only as compared to the untreated lead-intoxicated group but also compared with the normal control group. They also returned the mean value of nitric oxide toward its normal value. As for the MDA value, they induced a significant decrease compared with the untreated lead-intoxicated group. There were no noteworthy differences in the mean values of both MDA and NO in the kidney tissue homogenate (K. MDA and K. NO) between Roselle beverage-administered groups, while K. TAC was significantly greater in the CRB-administered group in comparison to the HRB-administered one (Table 3).

#### 4.2.3. Effect on Liver Functions

'The findings indicated that the activities of transaminases named alanine aminotransferase and aspartate aminotransferase (ALT and AST, respectively), ALP, and GGT were significantly (*P* < 0.05) higher in the untreated lead-intoxicated group compared with the normal control group. HRB administration could not affect the activities of these enzymes significantly as compared to the untreated lead-intoxicated group despite the noticed decrease. CRB was more efficient as it normalized the activities of transaminases and caused a significant decrease in GGT activity in comparison to the untreated lead-intoxicated group; however, the activity of ALP was not affected significantly. As for the total bilirubin concentration in serum, it was not affected significantly either by lead exposure or beverage consumption (Table 4).

On the other hand, the total protein and globulin concentrations in serum of the untreated lead-intoxicated group were found to be significantly (*P* < 0.05) lower than those of the normal control group. Both CRB and HRB induced no significant elevation as compared to the untreated lead-intoxicated group; however, CRB was the best. Regarding serum albumin, there were no notable distinctions among the four studied groups, although the decrease was noticed in the untreated lead-intoxicated group and the improvement was noticed in Roselle beverage-administered groups (Table 4).

#### 4.2.4. Effect on Kidney Functions and Electrolytes

The effects of Roselle calyx beverages on creatinine, urea, sodium, and potassium concentrations in serum of lead-intoxicated versus normal rats are illustrated in Table 5. Lead exposure alone led to a significant increase in serum creatinine compared to the normal control group, while the increase in the serum urea was insignificant. Lead exposure along with the consumption of cold tea induced a significant reduction in both creatinine and urea concentrations compared to the untreated lead-intoxicated group with no significant differences in comparison to the normal control group. While HRB consumption decreased serum creatinine significantly compared to the untreated lead-intoxicated group, it caused an insignificant decrease in serum urea level. Regarding sodium and potassium concentrations in serum, they were not affected significantly either by lead exposure or beverage consumption.

### 4.3. Effect on Liver and Kidney Histopathology

#### 4.3.1. Liver Histopathology

**Figure 2:**
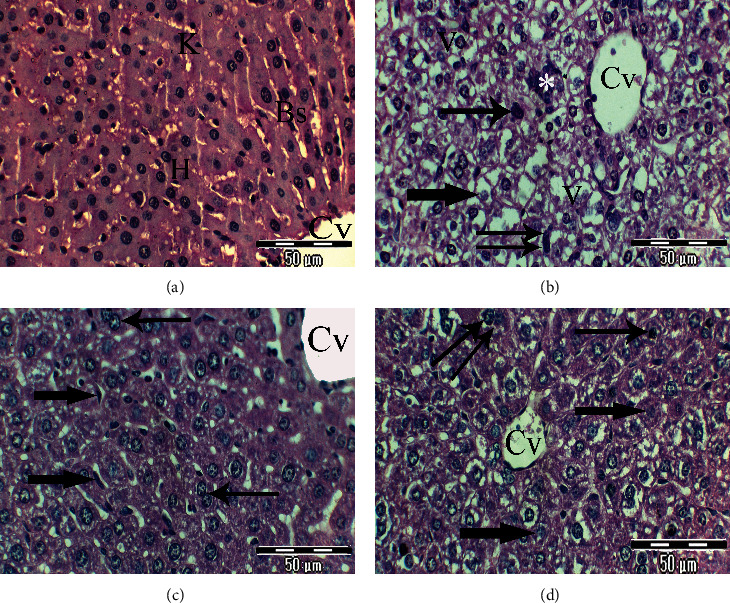
(a–d) Photomicrographs of rat liver sections of different experimental groups stained with Haematoxylin & Eosin. (a) Liver section of control rats showing normal hexagonal hepatic lobules; the central vein (Cv) is located in the central part. Hepatocytes (H) are organized into hepatic cords and separated by normal Kupffer (K) cells in blood sinusoids (Bs) (×400). (b) Liver section of rats from the untreated lead-intoxicated group showing a noticeable disorganized liver section, degenerative hepatocytes with a vacuolated cytoplasm (V) and demarcated membrane, dilated and widening central vein (Cv), noticeable cellular infiltration (∗), pronounced nuclear changes such as pyknotic nuclei (thin arrows) and karyolitic ones (thick arrows), and distinct phagocytic Kupffer cells (double arrows) (×400). (c) Liver section of rats from the CRB-administered group exhibits noticeable improvement of hepatic architecture, hepatic cords radiating from the normal central vein (Cv), normal appearance of most nuclei but increase in the number of binucleated hepatocytes (thin arrows), and regular blood sinusoid network with Kupffer cell activity (thick arrows) (×400). (d) Section of the rat liver from the HRB-administered group shows mild improvement of hepatic tissue, most of hepatocytes are intact, some had a degenerative and vacuolated cytoplasm, most of the nuclei are normal, and others are pyknotic (thin arrows), karyolitic (thick arrows), and megakaryocytic ones (double arrows) (×400).

The examination of haematoxylin- and eosin- stained liver sections of rats from the control (group 1) group showed normal architecture of the liver with hepatic lobulation. The hepatic strands are alternating with narrow blood sinusoids that are covered with endothelial layer cells having the Kupffer cells. The hepatocyte is polyhedral in shape and possesses a homogenously stained and granular cytoplasm. The hepatic cells have centrally located spherical nuclei, with a distinct nuclear envelope and one or more prominent nuclei, occasionally together with the number of chromatin granules. Some hepatic cells are binucleated (Figure 2(a)). Many histopathological alterations were seen in the liver sections of rats from the untreated lead-intoxicated group (group 2). They include cellular disorganization, hepatocyte degeneration especially in the centro-lobular zone and cytoplasmic vacuolation, pronounced nuclear changes, pyknotic nuclei and karyolitic ones, increase in Kupffer cell activity, and mononuclear infiltration (Figure 2(b)). Examination of liver sections of rats from the CRB-administered group (group 3) showed noticeable improvement of liver architecture, represented by the regular arrangement of hepatocytes around the central vein; their cytoplasm acquires its homogenous stain and granular appearance, but some hepatocytes appeared binucleated, and slight widening of blood sinusoids with activated Kupffer cells was observed (Figure 2(c)). Liver sections of rats from the HRB-administered group (group 4) showed mild improvement in the tissue and notable degree of restoration of its normal-like structures, represented by the regular arrangement of hepatocytes around the central vein, but some hepatocytes exhibit faintly stained vacuolated cytoplasm, pyknotic nuclei, and karyolitic and megakaryocytic ones (Figure 2(d)).

#### 4.3.2. Kidney Histopathology

**Figure 3:**
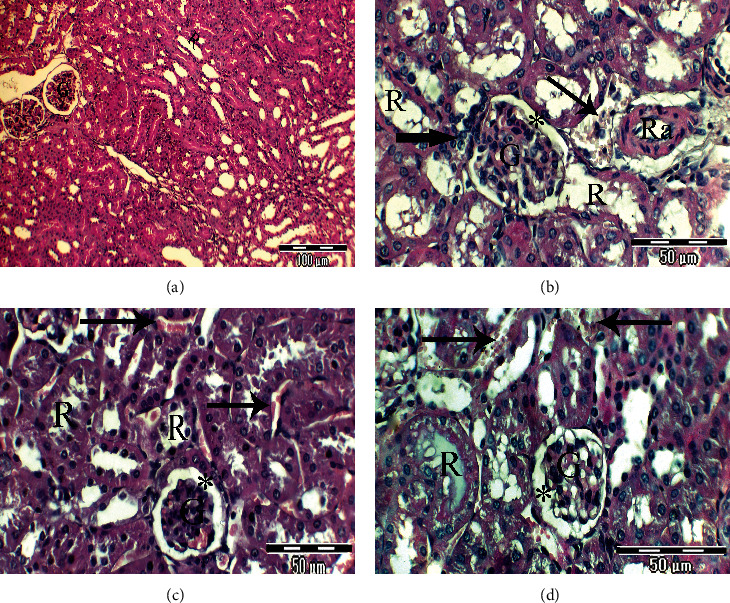
(a–d) Photomicrographs of rat kidney sections of different experimental groups stained with Haematoxylin & Eosin. (a) Renal cortex of rats in the control group has a normal body structure of renal glomeruli (G) and renal tubules (×200). (b) High-magnification view of a portion of the rat kidney from the untreated lead-intoxicated group exhibiting disorganized kidney anatomy, irregular glomeruli with irregular and atrophied mesangial areas (star), necrosis of renal tubules with atrophy and destruction of their lining epithelium (thick arrow), severe congestion of the renal vein, and fibrinoid necrosis of the renal artery (Ra) (×400). (c) High-magnification view of a portion of the rat kidney from the CRB-administered group exhibits the normal anatomy of most glomeruli and a mesangial section (star) that appears normal, elongated and distended renal tubules, and intertubular hemorrhage (arrows) (×400). (d) High-magnification view of a portion of the rat kidney from the HRB-administered group showing mild improvement of the kidney anatomy, glomeruli with an irregular mesangial area (star), atrophy of some renal tubules, others with hyaline casts, and intertubular hemorrhage (thick arrows) (×400).

Light microscopic examination of the renal cortex of rats from the control group (group 1) displayed a typical (normal) appearance. Bowman's capsule (mesangial area) surrounds the glomerulus, which is bordered by two layers of the epithelium. The glomeruli are round and oval in shape, the proximal convoluted tubules were lined with the simple cuboidal or columnar epithelium, its cells had an acidophilic cytoplasm, and the apex possesses abundant microvilli which formed a brush border. The distal convoluted tubules were lined with the simple cuboidal epithelium (Figure 3(a)). In Figure 3(b), a high-magnification view of a portion of the rat's kidney from the untreated lead-intoxicated group (group 2) showed disorganization of kidney anatomical appearance, irregular glomeruli with irregular and atrophied mesangial areas, necrosis of renal tubules with atrophy and destruction of their lining epithelium, severe congestion of the renal vein, and fibrinoid necrosis of the renal artery. In the CRB-administered group (group 3), a renal profile is nearly restoring its normal anatomical appearance. The majority of glomeruli had a regular oval or rounded shape with prominent mesangial areas, but irregular distended and dilated renal tubules and intertubular hemorrhage was seen (Figure 3(c)). On examination of the kidney of rats from the HRB-administered group (group 4), mild improvement of the kidney structures, glomeruli with the irregular mesangial area, atrophy of some renal tubules, others with hyaline casts, and severe intertubular hemorrhage were noticed (Figure 3(d)).

## 5. Discussion

The present findings indicated that a cold Roselle beverage was higher in both anthocyanin content and antioxidant activity than hot beverages. In this regard, it was found that both cold (25 °C for 240 min) and hot (90 °C for 16 min) aqueous extracts of Hibiscus sabdariffa L. calyces had two significant anthocyanins: delphynidin-3-sambubioside and cyanidin-3-sambubioside. Both extracts also were found to yield similar phytochemical properties [9]. Recently, response surface methodology was used to identify temperature (70–100°C), calyx to water ratio (1–20 g/100 ml), and time (1 to 30 minutes) that would produce a *Hibiscus* infusion with the highest total antioxidant activity and anthocyanin content. The best infusion was found to be prepared using 10 g dry calyces/100 ml at 88.7°C for 15.5 min [30]. In the present study, there was no difference between CRB and HRB regarding the used calyx to water ratio (1 : 40 weight/volume); however, the cold beverage was prepared at 25°C for 12 hr, while the hot beverage was prepared at 95°C for 15 min. Thus, it could be understood that the ratio of calyx to water showed no impact on this difference in total anthocyanin content and total antioxidant activity between the two studied beverages. Because anthocyanins are the main antioxidant chemicals in Hibiscus calyces, a long duration of extraction appears to be beneficial in extracting more anthocyanin and thus obtaining a beverage with increased antioxidant activity. At the same time, temperatures above 88.7°C were reported to decrease total anthocyanin content in the resulted infusions [30], which means that anthocyanins degraded at higher temperatures.

In the present study, lead exposure resulted in overweight. This finding was in line with the results of several human and animal studies. For example, it was reported that there was something good (positive) about the association between blood lead level and BMI in women, but not in men (Chinese people) [1]. In an animal study, the metabolic disorders associated with chronic exposure of adult rats to lead toxicity, including insulin insensitivity and weight gain, may be resulting from altered methylation of metabolism-related genes [31].

Conversely, the weight loss-promoting impact of *Hibiscus sabdariffa* L. calyx beverages (CRB and HRB) was reported from many previous studies. In this regard, it was revealed that oral *Hs* aqueous extract administration (150, 200, 250, and 300 mg/kg) for 10 weeks caused a dose-dependent reduction in BWG and abdominal fats in obese rats [32]. The mechanisms by which *Hs* can induce weight loss were investigated. Among them, the abilities of *Hs* to suppress appetite and inhibit the activities of carbohydrate digestive enzymes including *α*-/*β*-glucosidase and *α*-amylase were the most considered [32, 33].

In the present study, it seems like that the increased body weight of the untreated lead-exposed group was not accompanied by a similar increase in its liver weight; i.e., liver weight remained stable. So, the relative liver weight was significantly lower than that of the normal control group. This finding can be attributed to the ability of lead toxicity to inhibit protein synthesis [34, 35].

Regular exposure to lead acetate, in our study, led to a significant (*P* < 0.05) rise in tissue Pb. These findings were in agreement with the results of Abdel Moneim et al. [13, 36]. On the other hand, the reduction of ALAD activity in liver and kidney tissue homogenates as a result of Pb exposure was supported by Amin et al. [37]. It is well known that lead inhibits the activities of ferrochelatase, aminolevulinic acid (ALA) synthetase, and ALAD, the three enzymes that are important for the heme biosynthetic pathway; however, the inhibitory impact is stronger on ALAD enzyme activity. So, the inhibition of ALAD activity is an indicator for determining the lead poisoning severity. ALAD has a sulfhydryl moiety that makes it vulnerable to cause attacks. Lead makes ALAD's activity come to a halt, leading to the accumulation of aminolevulinic acid (ALA); this in turn causes the reactive oxygen species (ROS) production [38], which attack cell membranes and oxidize its bioactive compounds, leading to the reduction of total antioxidant capacity and hence elevation of lipid peroxidation product “MDA.” This accounts for the present results markedly. In agreement, El-Tantawy [39] reported that Pb exposure dysregulated antioxidant/oxidant balance in the liver tissue homogenate. Similarly, lead exposure was found to cause renal cytotoxicity mainly by lowering the activities of antioxidant enzymes as well as rising reactive oxygen species and lipid peroxidation [13]. Pb intoxication was reported to induce lipid peroxidation indirectly via damaging the protective antioxidant barrier which in turn happens through binding to thiol groups of antioxidant enzymes [38].

The lowering effect of lead acetate administration on nitric oxide level in liver and kidney tissue homogenates, as indicated by the present results, was in harmony with Garcia-Arenas et al. [40] who revealed that Pb2+ interferes with NO production as a result of its chemical similarity with Ca2+. In more detail, Pb2+ hinders the type I nitric oxide synthase (NOS) which is Ca2+- and calmodulin-dependent and produces NO constitutively (cNOS) [41].

The harmful effects of lead acetate on liver functions evidenced by the substantial increase in the activities of serum transaminases, as revealed by the present results, can be attributed to the fact that Pb2+ accumulation enhanced the production of free radicals which in turn destroy liver cells leading to the release of liver enzymes into the circulation. In support, El-Tantawy [39] found that the levels of MDA and reactive oxygen species, as well as the activities of transaminases, were increased significantly in the lead-treated group, while superoxide dismutase and reduced glutathione activities showed a significant decline.

As for ALP, it is present in the intra- and extrabiliary duct walls, and its elevation may express damage of the biliary cells [42]. GGT, however, is often located in hepatocyte membranes and has many functions including the transport of peptides and amino acids into the cell as *γ*-glutamyl peptides. It may also be embarrassed in glutathione metabolism, and its increase is itself a marker of oxidative stress [43].

On the other hand, binding to plasmatic proteins, lowering hepatic DNA and RNA, and decreasing the utilization of free amino acids for protein synthesis in hepatocytes are three possible mechanisms by which Pb exposure induces a hypoproteinemic effect [34, 35].

In general, the increased activities of liver enzymes (ALP, ALT, AST, and GGT) in serum along with the decrease in serum total protein as a result of lead exposure are supported by Moussa and Bashandy [35].

In the present study, lead exposure caused hypercreatininemic effect, while it caused an insignificant increase in serum urea. These results are in harmony with animal and human studies [13, 36] and can be attributed to increased oxidative stress in renal tissue as a result of lead toxicity [13]. Moreover, the untreated lead-exposed group showed insignificant reductions in serum sodium and potassium compared with the normal control group. These results are in accordance with those of Amah et al. [36]; the poor sensitivity of electrolytes as indicators for kidney function as well as the renal reserve capability to maintain normal function even after the loss of about 50% of nephrons can account for these findings.

In general, the histopathological alterations noticed in liver and kidney tissues of the untreated lead-intoxicated group, in the current research, are in agreement with the results of many studies [34, 44]. The capability of lead to enhance ROS generation resulted in oxidative damage in many tissues evidenced by decreased total antioxidant capacity, and lipid peroxidation elevation, as shown formerly, is the mechanism by which lead causes these morphological abnormalities in liver and kidney tissues, as suggested in many studies [13, 39].

According to the present results, Roselle beverages, especially the cold one, prevented the consequences of Pb toxicity. This was evidenced by decreasing Pb accumulation as well as oxidative markers in liver and kidney tissues and lowering their structure abnormalities and insufficiency markers. The hepatorenal protective properties of the tested Roselle beverages were in harmony with the results of many studies in which these effects were attributed to the antioxidant capacity evidenced by increasing antioxidant enzyme activities and decreasing lipid peroxidation in induced models of liver and kidney injuries [45, 46].

In fact, studies on the effect of *Hs* on Pb-induced toxicity are scarce. However, the antioxidant effects of *Hs* extracts in the face of many other hepatorenal toxic agents were investigated. One of them is cadmium (Cd), a heavy metal like lead in its toxic effect. It was reported that exposure to Cd, for fifteen days, led to a significant decrease (*P* < 0.05) in reduced glutathione levels and the activities of antioxidant enzymes including superoxide dismutase, glutathione-S-transferase, and catalase in liver and kidney tissues significantly (*P* < 0.05) elevated lipid peroxidation in the plasma and tissues of rats. On the other hand, pretreatment with *Hs* anthocyanins improved the activities of the antioxidant enzymes in tissues, with concomitant increase in reduced glutathione levels and decrease in tissue lipid peroxidation markers [47]. *H. sabdariffa* antioxidant activity may be credited with its high content of phenolic aromatic compounds [48]. Studies on the antioxidant effect of herbs confirm the therapeutic potential properties of polyphenols [49]. Protocatechuic acid, an aromatic compound, found in the *Hs* calyces has been identified to have a hepatoprotective effect [50]. However, the compounds which are reported to be mainly responsible for these therapeutic effects of *Hs* are anthocyanins [45, 51]. Accordingly, the higher content of total anthocyanins and total antioxidant activity of the cold beverage, compared to the hot one, elucidated its efficiency in alleviating the hepatorenal abnormalities resulting from Pb exposure.

## Figures and Tables

**Table 1:** Total content of anthocyanin content and antioxidant activities of Roselle beverages.

Parameters	CRB	HRB
Total anthocyanin content (mg C3G/100 ml)	167.32	100.83
Total antioxidant activity (*μ*mol TE/100 ml)	1270.11	780.20

CRB: cold Roselle beverage; HRB: hot Roselle beverage; C3G: cyanidin-3-glucoside equivalents; TE: equivalents.

**Table 2:** Effects of Roselle red calyx beverages on BWG and absolute RLW and RKW in lead-intoxicated versus normal rats.

Parameters	Normal control	Lead-intoxicated	Lead-intoxicated+CRB	Lead-intoxicated+HRB	*P* value
Initial weight (g)	158.33 ± 15.28	161.67 ± 20.21	151.67 ± 10.41	166.33 ± 12.66	0.494
Final weight (g)	221.60 ± 23.33^ab^	249.30 ± 16.43^c^	216.07 ± 7.18^a^	239.86 ± 3.77^bc^	0.009
BWG (g)	63.27 ± 8.05^a^	87.63 ± 3.78^c^	64.40 ± 3.23^a^	73.53 ± 8.89^b^	<0.001
LW (g)	7.11 ± 0.98	7.02 ± 0.76	6.59 ± 0.83	7.01 ± 0.75	0.763
RLW (%)	3.20 ± 0.11^b^	2.81 ± 0.12^a^	3.04 ± 0.28^ab^	2.92 ± 0.27^ab^	0.053
KW (g)	1.33 ± 0.25	1.60 ± 0.24	1.50 ± 0.23	1.38 ± 0.23	0.307
RKW (%)	0.60 ± 0.05^ab^	0.64 ± 0.05^ab^	0.69 ± 0.08^b^	0.57 ± 0.09^a^	0.080

Results are expressed as mean ± SD. Significance level at *P* < 0.05. Similar letters are implying partial or complete nonsignificance. CRB: cold Roselle beverage; HRB: hot Roselle beverage; BWG: body weight gain; LW: liver weight; RLW: relative liver weight; KW: kidney weight; RKW: relative kidney weight.

**Table 3:** Effects of Roselle red calyx beverages on lead, ALAD, and oxidative stress markers in liver and kidney tissues of lead-intoxicated versus normal rats.

Parameters	Normal control	Lead-intoxicated	Lead-intoxicated+CRB	Lead-intoxicated+HRB	*P* value
L. Pb (*μ*g/g tissue)	0.42 ± 0.05^a^	3.10 ± 0.39^d^	1.24 ± 0.13^b^	2.44 ± 0.30^c^	<0.001
L. ALAD (U/mg protein)	0.80 ± 0.10^d^	0.20 ± 0.03^a^	0.60 ± 0.08^c^	0.32 ± 0.04^b^	<0.001
L. MDA (nmol/mg)	0.14 ± 0.03^a^	0.24 ± 0.05^b^	0.15 ± 0.03^a^	0.21 ± 0.04^b^	0.001
L. NO (*μ*mol/mg)	0.19 ± 0.02^b^	0.14 ± 0.03^a^	0.17 ± 0.03^ab^	0.14 ± 0.02^a^	0.018
L. TAC (ng/ml)	0.31 ± 0.04^b^	0.15 ± 0.03^a^	0.28 ± 0.04^b^	0.16 ± 0.03^a^	<0.001
K. Pb (*μ*g/g tissue)	0.57 ± 0.06^a^	4.88 ± 0.65^c^	2.23 ± 0.27^b^	2.56 ± 0.30^b^	<0.001
K. ALAD (U/mg protein)	0.37 ± 0.05^d^	0.06 ± 0.01^a^	0.30 ± 0.04^c^	0.22 ± 0.02^b^	<0.001
K. MDA (nmol/mg)	0.10 ± 0.01^a^	0.36 ± 0.03^c^	0.22 ± 0.02^b^	0.24 ± 0.04^b^	<0.001
K. NO (*μ*mol/mg)	0.19 ± 0.02^b^	0.10 ± 0.02^a^	0.19 ± 0.02^b^	0.17 ± 0.02^b^	<0.001
K. TAC (ng/ml)	0.15 ± 0.02^b^	0.11 ± 0.02^a^	0.21 ± 0.02^d^	0.18 ± 0.02^c^	<0.001

Results are expressed as mean ± SD. Significance level at *P* < 0.05. Similar letters are implying partial or complete nonsignificance. CRB: cold Roselle beverage; HRB: hot Roselle beverage; L. Pb: liver lead; L. ALAD: liver delta-aminolevulinic acid dehydratase; L. MDA: liver malondialdehyde; L. NO: liver nitric oxide; L. TAC: liver total antioxidant capacity; K. Pb: kidney lead; K. ALAD: kidney delta-aminolevulinic acid dehydratase; K. MDA: kidney malondialdehyde; K. NO: kidney nitric oxide; K. TAC: kidney total antioxidant capacity.

**Table 4:** Effects of Roselle red calyx beverages on liver functions in serum of lead-intoxicated versus normal rats.

Parameters	Normal control	Lead-intoxicated	Lead-intoxicated+CRB	Lead-intoxicated+HRB	*P* value
AST (U/l)	151.25 ± 13.92^a^	186.33 ± 11.12^c^	159.00 ± 17.59^ab^	178.00 ± 15.12^bc^	0.005
ALT (U/l)	123.20 ± 19.18^a^	172.80 ± 24.54^b^	127.00 ± 17.68^a^	160.80 ± 25.38^b^	0.005
ALP (U/l)	181.67 ± 13.06^a^	220.00 ± 18.38^b^	196.00 ± 16.08^ab^	200.20 ± 21.61^ab^	0.025
GGT (U/l)	20.00 ± 3.16^a^	31.33 ± 3.34^c^	25.60 ± 3.83^b^	27.00 ± 2.93^bc^	0.001
Total bilirubin (mg/dl)	0.75 ± 0.10	0.80 ± 0.12	0.75 ± 0.12	0.76 ± 0.13	0.880
Total protein (g/dl)	8.22 ± 1.29^b^	6.66 ± 0.65^a^	7.22 ± 0.77^ab^	6.84 ± 0.84^a^	0.070
Albumin (g/dl)	4.08 ± 0.79	3.56 ± 0.27	3.58 ± 0.22	3.54 ± 0.39	0.246
Globulin (g/dl)	4.14 ± 0.51^b^	3.10 ± 0.43^a^	3.64 ± 0.40^ab^	3.30 ± 0.57^a^	0.019

Results are expressed as mean ± SD. Significance level at *P* < 0.05. Similar letters are implying partial or complete nonsignificance. CRB: cold Roselle beverage; HRB: hot Roselle beverage; ALP: alkaline phosphatase; ALT: alanine aminotransferase; AST: aspartate aminotransferase; GGT: gamma-glutamyltransferase.

**Table 5:** Effects of Roselle red calyx beverages on kidney functions and electrolytes in serum of lead-intoxicated versus normal rats.

Parameters	Normal control	Lead-intoxicated	Lead-intoxicated+CRB	Lead-intoxicated+HRB	*P* value
Creatinine (mg/dl)	0.82 ± 0.11^a^	1.10 ± 0.16^b^	0.72 ± 0.10^a^	0.87 ± 0.13^a^	0.002
Urea (mg/dl)	46.60 ± 6.35^ab^	56.00 ± 8.89^b^	44.75 ± 5.99^a^	50.80 ± 8.44^ab^	0.127
Na (mmol/L)	138.39 ± 12.91	136.31 ± 14.92	138.33 ± 11.37	136.53 ± 12.88	0.990
K (mmol/L)	3.97 ± 0.50	3.57 ± 0.46	4.10 ± 0.43	3.60 ± 0.46	0.219

Results are expressed as mean ± SD. Significance level at *P* < 0.05. Similar letters are implying partial or complete nonsignificance. CRB: cold Roselle beverage; HRB: hot Roselle beverage.

## Data Availability

All relevant data supporting the findings of this study are available within the article.
